# Patient-Derived Organoids from Pancreatic Neuroendocrine Tumors: A Systematic Review of PDO Take Rates, Molecular–Biological Characteristics, and Potential for Clinical Utility

**DOI:** 10.3390/cancers17203364

**Published:** 2025-10-18

**Authors:** Celine Oanæs, Marcus T. T. Roalsø, Marina Alexeeva, Kjetil Søreide

**Affiliations:** 1 Gastrointestinal Translational Research Unit, Stavanger University Hospital, 4019 Stavanger, Norway; celine.oanes@sus.no (C.O.); marcus.t.roalso@uis.no (M.T.T.R.); marina.alexeeva@sus.no (M.A.); 2 HPB Unit, Department of Gastrointestinal Surgery, Stavanger University Hospital, 4019 Stavanger, Norway; 3 Department of Quality and Health Technology, University of Stavanger, 4021 Stavanger, Norway; 4 Department of Clinical Medicine, University of Bergen, 5020 Bergen, Norway

**Keywords:** pancreatic neuroendocrine neoplasm, PanNEN, neuroendocrine tumor, NET, neuroendocrine carcinoma, NEC, patient-derived organoid, PDO, tumoroid, spheroid

## Abstract

**Background:** Pancreatic neuroendocrine neoplasia (PanNEN) comprises a spectrum, from well-differentiated (i.e., G1, G2) pancreatic neuroendocrine tumors (PanNETs) to poorly differentiated carcinomas (PanNECs). Therapeutic progress is limited by the lack of representative preclinical models. Patient-derived organoids (PDOs) offer potential as translational models, but evidence remains scattered. **Methods:** We conducted a systematic review of PubMed (Jan 2009–Aug 2025) for original studies reporting on PDOs from PanNEN patients. Eligible studies were screened using the Rayyan software and data extracted from PDO take rates, validation methods, and clinical applications. **Results:** Twelve studies were included for qualitative and quantitative analyses. PDOs were successfully generated from both PanNETs (G1–G3; *n* = 26) and PanNECs (*n* = 6), primarily derived from primary tumors, but several studies also included metastatic sites. Take rates ranged from 33% to 100%, for a cumulative 33 PDOs from 44 attempts (overall take rate: 75%). Validation consistently employed histology, immunohistochemistry, and molecular profiling, with several studies incorporating xenotransplantation or omics approaches. PDOs demonstrated variable culture durations, from short-term (<3 weeks) to long-term (>20 passages). Drug screening studies (*n* = 7) revealed heterogenous responses to standard agents and pathways (everolimus, sunitinib, and temozolomide) and identified novel vulnerabilities, including EZH2 dependency, PI3K/CDK4/6 synergy, and Bcl-2-linked sensitivities in PanNECs. One study provided evidence of concordance between PDO drug sensitivity and patient responses. **Conclusions:** Research into PanNEN organoids remains limited. However, PDOs can preserve key histological and molecular features, enable pharmacotyping, and uncover candidate biomarkers for therapy. Despite feasibility across subtypes, progress is constrained by variability in culture success. Standardization and prospective validation are essential to advance PDOs as tools for personalized medicine in PanNENs.

## Simple Summary

Pancreatic neuroendocrine neoplasia (PanNEN) consists of a set of rather rare and heterogeneous tumors, spanning from well-differentiated pancreatic neuroendocrine tumors (PanNETs) to poorly differentiated carcinomas (PanNECs). Therapeutic development has been limited by a lack of representative preclinical models. Patient-derived organoids (PDOs) represent a promising approach, preserving both histological and molecular features of the original tumors. In this systematic review, we summarize all published studies on PanNEN PDOs, including establishment success, validation strategies, culture longevity, and applications in drug testing. PDOs were successfully generated from both PanNETs and PanNECs, though take rates and culture durability varied. Drug screening studies highlighted inter-patient heterogeneity and uncovered potential novel therapeutic targets. Despite their promise, challenges such as variable culture success and limited tissue availability underscore the need for standardized protocols and prospective validation to fully realize their translational potential.

## 1. Introduction

Gastroenteropancreatic neuroendocrine neoplasia (GEP-NEN) is increasing in incidence [1,2,3], with the most common location of GEP-NENs being in the small bowel and the pancreas [1,2,4]. Pancreatic neuroendocrine neoplasms (PanNENs) are generally rare malignancies, representing about 2% of all pancreatic tumors [5]. While PanNENs can occur on a hereditary background based on familial genetic syndromes such as multiple endocrine neoplasia type 1 (MEN1) or von Hippel–Lindau disease (VHL), of which some may also be found in sporadic cases [6], most genomic events are sporadic and associated with specific genetic and epigenetic events in at least four main pathways [6,7,8,9,10].

PanNENs comprise a spectrum, from well-differentiated pancreatic neuroendocrine tumors (PanNETs) to poorly differentiated pancreatic neuroendocrine carcinomas (PanNECs) [7]. Histological grading (G1–G3) reflects differentiation and proliferative activity [11,12], correlating closely with prognoses, where the 5-year OS rates in G1, G2, and G3 are 77%, 63%, and 20%, respectively [11,13]. Management of PanNEN is controversial across the spectrum of the disease, with numerous treatment options available [4,14,15,16,17,18], and ranges from simple surveillance of small, non-functional tumors to routine surgical resection for functional PanNETs presenting with symptoms. Approximately 90% of PanNENs are low-grade PanNETs, whereas PanNECs form a smaller but more aggressive subset with limited therapeutic options and poor outcomes [19,20].

PanNETs can be divided into functional and non-functional (NF) groups, with the latter being most prominent. Functional PanNETs present hormone-related symptoms from the hormonal hypersecretion of the tumor, allowing for early diagnosis. NF-PanNETs often remain asymptomatic until late stages with advanced disease [21] or present as ‘insidentalomas’ on imaging performed for other indications.

The clinical course of PanNETs is heterogenous, with variable progression rates and responses to therapy. For patients with localized PanNETs, surgical removal serves as the primary and only potentially curative option [17]. With unresectable or metastatic disease [22], first-line medical therapy typically involves somatostatin analogs (SSAs) [23]. Second-line treatments may include targeted agents (everolimus and sunitinib) [24,25,26,27], chemotherapy (temozolomide and streptozocin) or peptide receptor radionuclide therapy (PRRT) [28,29]. However, responses are often transient, and no robust biomarkers exist to guide their use. Several controversial areas with little evidence to guide decisions also exist for panNENs, particularly in the metastatic setting [30,31].

Advancing therapy development for PanNENs has been hindered by the limited availability of preclinical models that accurately capture the biology of these tumors. BON-1 and QGP-1 cells are among the most commonly used commercially available 2D cell lines for in vitro studies of pancreatic neuroendocrine tumors [32]. However, conventional cell lines fail to capture the biological complexity of patient tumors, and patient-derived xenografts (PDXs) are costly [33]. Patient-derived organoids (PDOs) offer an attractive alternative [34,35,36]. These three-dimensional cultures preserve histological and molecular fidelity, can be expanded from limited tissue, and have proven as a representative model for drug screening and translational studies in other cancer types [36,37,38,39].

Although recent studies have been able to show the feasibility of establishing patient-derived organoids from tissue samples from patients with PanNENs, the current published work is scattered and lacks systematic consolidation to assess the overall reported success rates and potential for clinical application [40,41,42]. Individual studies have described PanNET and PanNEC organoids, but no focused synthesis has clarified their take rates, molecular characterization, or use in therapeutic testing. Here, we present a systematic review of organoids derived specifically from pancreatic neuroendocrine neoplasms, with the goal of consolidating current evidence and identifying future opportunities for preclinical modeling in this rare but clinically challenging disease spectrum.

## 2. Methods

The study follows the guidelines provided by the Preferred Reporting Items for Systematic Reviews and Meta-Analyses (PRISMA) 2020 statement [43]. The PICO elements are described as follows:

Population: Patients with pancreatic neuroendocrine neoplasms (G1–G3), with patient-derived organoids generated, were included.

Intervention: It involved the establishment of PanNEN-derived organoids for research or clinical applications.

Comparison: No comparison to PDO was used in this study. Where available, groups with successful PDO growth were compared to those with no growth.

Outcome: Variations in PDO take rates, validation methods, characterization, drug screening, and clinical correlations were determined.

### 2.1. Search Strategy

A systematic literature search was conducted in PubMed. The following search terms were used in our search: (“Pancreatic Neoplasms”[Mesh] OR pancreas[tiab] OR pancreatic[tiab] OR GEP[tiab] OR gastroenteropancreatic[tiab]) AND (“Neuroendocrine Tumors”[Mesh] OR “Insulinoma”[Mesh] OR “Gastrinoma”[Mesh] OR “Glucagonoma”[Mesh] OR “neuroendocrine neoplasm*”[tiab] OR “neuroendocrine tumor*”[tiab] OR “neuroendocrine carcinoma*”[tiab] OR NET[tiab] OR NETs[tiab] OR NEN[tiab] OR NENs[tiab] OR NEC[tiab] OR NECs[tiab] OR pNET[tiab] OR PanNET[tiab] OR pNEN[tiab] OR PanNEN[tiab] OR pNEC[tiab] OR PanNEC[tiab] OR “islet cell tumor*”[tiab] OR “islet cell carcinoma*”[tiab] OR carcinoid[tiab] OR insulinoma[tiab] OR gastrinoma[tiab] OR glucagonoma[tiab]) AND (“Organoids”[Mesh] OR organoid*[tiab] OR tumoroid*[tiab] OR spheroid*[tiab] OR “patient derived organoid*”[tiab] OR PDO[tiab] OR PDOs[tiab]). As the first report on 3D cultures derived from adult stem cells was published in 2009 [44], the literature search was accordingly confined to studies published from 2009 onwards. The systematic literature search was performed on 6 August 2025.

### 2.2. Inclusion Criteria

Given the limited number of publications in this emerging field, stringent inclusion and exclusion criteria were avoided to maintain a broader search scope. Studies were eligible if they met all the following conditions:

1. Reported the establishment or use of PDOs generated from PanNENs, including PanNET (G1–G3) or PanNECs.
2. Were original research articles published in peer-reviewed journals, with the full text available in English.
3. Were published between 1 January 2009, and 6 August 2025.

### 2.3. Exclusion Criteria

Studies were excluded if they met any of the following conditions:

1. Used non-human or non-patient-derived material (e.g., established cell lines, xenografts, or animal models only).
2. Did not involve established organoids.
3. Did not report PanNENs (e.g., solely exocrine pancreatic tumors or other GEP-NENs).
4. Were reviews, editorials, conference abstracts, or preprints, without peer review.
5. Lacked sufficient methodological details or relevant outcome data related to PDO establishment, validation, or other applications.

### 2.4. Data Extraction

The results of the literature search were imported into Rayyan (https://www.rayyan.ai), an AI-assisted tool designed to facilitate systematic reviews [45]. An initial screening was conducted based on titles and abstracts, followed by a full-text review in the final screening round. Two independent reviewers screened articles for eligibility (C.O. and M.A.) and initially agreed on 92.7% of the studies. Any disagreements were resolved through discussion, and if not, they were handled by a third reviewer (M.T.T.R). Studies that met the inclusion criteria were selected, and relevant data were extracted using a standardized data-extraction table. The extracted variables included the take rate of PDO establishment, validation, and characterization methods, as well as the potential for clinical translation in therapeutic applications.

### 2.5. Quality Appraisal and Risk-of-Bias Consideration

The studies in this review were highly heterogenous, focusing on establishment, characterization, and potential for therapeutic applications, rather than interventional comparisons. As such, formal risk-of-bias assessment tools were deemed not applicable. Instead, a qualitative appraisal of methodological transparency was performed, emphasizing clarity of tumor source description, take rate, culture duration, and validation and characterization methods. This narrative approach aligns with recommendations for systematic reviews of model-development studies where standardized bias frameworks are unsuitable.

## 3. Results

**Figure 1:**
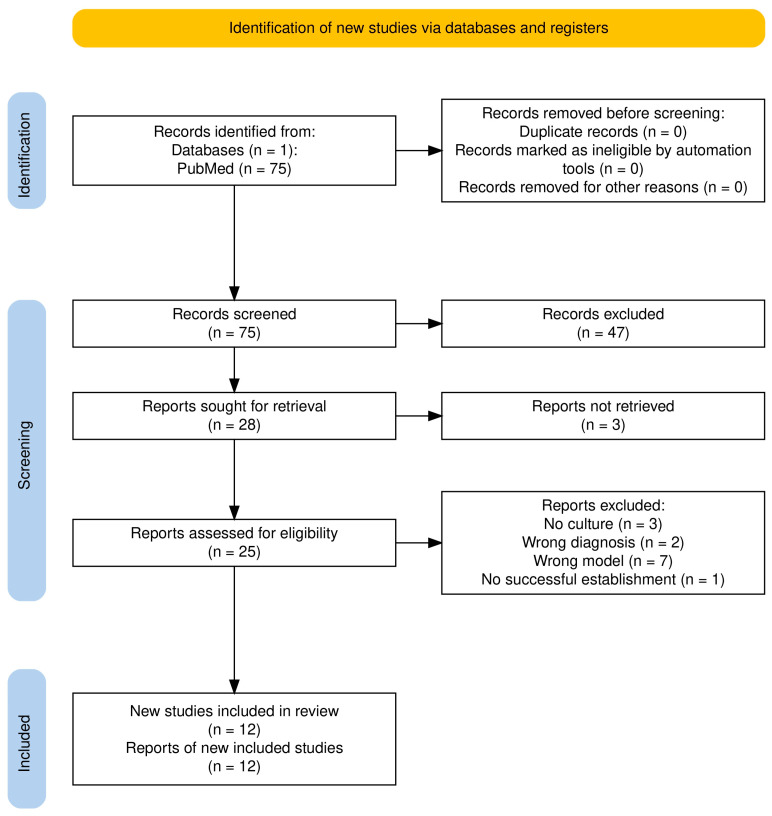
PRISMA flowchart of the article screening process [43].

The search identified 75 records for further screening. Of the 75 identified records, no duplicates were found, and they were all included in the screening process. After screening titles and abstracts and applying the exclusion criteria, a total of 25 articles remained for full-text retrieval, of which 12 met the inclusion criteria (Figure 1).

The included studies were all published between 2020 and 2025, reflecting the recent emergence of this field. Five studies appeared between 2020 and 2022, while the majority (*n* = 7) were published from 2023 onward (Table 1). Studies were predominantly European (*n* = 8), with additional contributions from Asia (*n* = 3) and North America (*n* = 2).

In addition to publication year and geography, the included studies differed in their objectives and scope. Some focused on establishing a single PDO line to test methodological innovations, whereas others aimed to systematically generate biobanks or compare a broader spectrum of NENs, such as GEP-NENs. For this review, data were extracted exclusively for organoids established from PanNEN samples.

### 3.1. Current Overview of Patient-Derived Organoid Models in PanNENs

Across the 12 studies [21,46,47,48,49,50,51,52,53,54,55,56], PDOs were generated from both PanNETs and PanNECs (Table 2). Six studies reported PanNET PDOs exclusively, including three that specifically described NF-PanNET PDOs [21,48,55]. Four studies included both PanNET and PanNEC samples; in one of these, PanNETs could not be established, leaving only PanNEC PDOs [46]. Two studies reported PanNEC PDOs exclusively [50,51], although these publications describe the same underlying PanNEC PDO line, with Alcala et al. [50] providing extended molecular characterization. In total, the studies report 26 successfully established PanNET PDOs and only 6 PanNEC PDOs. Among studies that reported the tumor grade, PDOs were derived across the spectrum of PanNETs, including one study on G1 tumors, five on G2 tumors, and five on G3 tumors.

PDOs were derived from both primary tumors and metastatic lesions. Primary tumors represented the most common source, while several studies also reported successful derivation from liver [47,49,52,53,55] and lymph node metastases [49]. This demonstrates feasibility across different anatomical sites, though with variable efficiency.

### 3.2. Validation and Characterization Methods

All included studies validated PDO identity and fidelity against the corresponding patient tumor tissue using combinations of histology, immunohistochemistry (IHC), and molecular profiling (Table 2). Commonly used IHC markers included synaptophysin (SYN) and chromogranin A (CGA), with CD56 and insulinoma-associated protein 1 (INSM1) applied in some studies, all serving as neuroendocrine cell markers. Ki-67 was assessed as a proliferation marker.

For genetic fidelity, several approaches were used (Table 2). Short tandem repeat (STR) profiling was employed in two studies to authenticate sample identity and exclude cross-contamination [46,47]. Whole-exome sequencing (WES) was applied in two studies [21,48], providing detailed mutational concordance, and whole-genome sequencing (WGS) was performed in three studies [50,51,56], offering extensive genomic coverage, including structural variants and copy-number alterations. One study used the targeted mutational sequencing panel “Illumina TruSight Oncology 500 (TSO500) [49]. A total of six studies also incorporated RNA sequencing (RNA-seq) to assess transcriptomic fidelity and pathway activation [21,49,50,51,56], with one applying single-cell RNA-seq to capture intra-tumoral heterogeneity [48].

Beyond conventional methods, several groups integrated more advanced techniques. Ji et al. [21] combined proteomic profiling with PDO cultures to identify prognostic signatures, while Kawasaki et al. [56] implemented ATAC-seq and methylation arrays to define transcription factor-driven subtypes. Wang et al. [48] further demonstrated that PDOs preserved the expression of therapeutic targets such as SSTR2, the SSA treatment target, and VEGF, closely mirroring the patient tumors and supporting their utility for testing VEGF- and mTOR-directed therapies. Functional validation included xenotransplantation [47,48,51,56] and assays such as optical metabolic imaging [54], supporting the biological relevance of these models.

### 3.3. Feasibility of Patient-Derived Organoids in Pancreatic NENs

Only three studies reported failed attempts of PanNEN organoid establishment (Table 2). Nine studies reported 100% success, while the take rates of Roalsø et al. [46], April-Monn et al. [55], and Kawasaki et al. [56] were 33%, 86%, and 38%, respectively. The failed PDO attempts reported were exclusively from primary tumors and included two PanNETs (not graded), five PanNET G2, and one PanNEC, underscoring challenges of establishing organoids from certain samples.

The PDOs exhibited divergent growth characteristics. Several studies documented short-term cultures (<3 weeks) with limited passages [21,46,49,52,53,55], whereas others achieved long-term propagation, with some lines maintained beyond 6 months and >20 passages [47,56]. Alcala et al. [50] and Dayton et al. [51] demonstrated long-term culture of a PanNEC PDO line for more than a year. This highlights that, while long-term propagation is achievable in some cases, challenges remain in PDO establishment and in maintaining cultures over time.

Culture protocols varied across the studies. While most studies used fresh tumor tissue, April-Monn et al. [55] demonstrated the feasibility of generating PanNET PDOs from cryopreserved tissue. Digestion methods varied, from short Liberase or TrypLE treatments to longer collagenase digestions. Culture formats included embedding single cells directly into domes or seeding in ultra-low attachment plates to promote spheroid formation. Media composition differed, with some studies using Wnt3A, R-spondin, and Noggin. Strnadel et al. [47] attempted to adapt the culture protocol to better recapitulate the tumor microenvironment in vivo by dividing the tumor tissue in two and using the other half as a “feeder layer” for the PDOs. Altogether, these factors impact the take rate, growth dynamics, and phenotype preservation.

### 3.4. Functional Applications and Drug Screening

Seven studies reported drug screening efforts in PanNEN organoids (Table 3). Early reports tested clinically approved agents such as everolimus, sunitinib, and temozolomide [54,55], revealing variable PDO sensitivities that mirrored the clinical heterogeneity observed in patients. April-Monn et al. [53] extended this work by linking high EZH2 expression with sensitivity to an EZH2 inhibitor, illustrating the potential of PDOs for biomarker discovery.

Combination approaches also emerged. Gulde et al. [52] demonstrated the synergistic activity of PI3K (buparlisib) and CDK4/6 (ribociclib) inhibitors, while April-Monn et al. [49] identified a novel interaction between KDM5A/IFNB1 activation and a cisplatin response corresponding to a clinical response. Ji et al. [21] applied proteomic-guided drug screening in NF-PanNET PDOs, identifying subtype-specific vulnerabilities.

Notably, PDOs from PanNECs were also applied for pharmacotyping. Dayton et al. [51] showed that high ASCL1 expression predicted sensitivity to Bcl-2 inhibitors, while sensitivity to FK866 highlighted metabolic vulnerability in the neuroendocrine phenotype. These findings illustrate how PDOs may expand therapeutic avenues in aggressive PanNECs, where treatment options are particularly scarce.

Collectively, drug screening studies demonstrate that PDOs recapitulate patient-specific heterogeneity, enable preclinical testing of standard and investigational agents, and can reveal novel biomarker-drug associations with translational potential. Importantly, while most reports remained at the preclinical level, April-Monn et al. [49] provided the first direct evidence of concordance between PDO drug sensitivity and patient responses to cisplatin, underscoring the promise of PDOs as a platform for personalized therapy in PanNENs.

## 4. Discussion

This systematic review synthesizes the literature on PDOs in PanNENs. PDOs have now been established across multiple centers from PanNETs, confirming feasibility in both primary and metastatic settings. Notably, the overall number of studies remains limited, reflecting both the rarity of PanNENs and, most likely, also the technical challenges of generating durable PDO cultures from patient material [40,57]. While the first pancreatic NET cell line was established in 1980, the first PanNEN PDO was established and reported in 2020 [56], making this technique less than 5 years old since its first success and hence indicating a limited time from which further studies could have been derived. Of note, similar models for pancreatic cancer has only been around for a decade [58]. Hence, as also noted by others, the current preclinical models for investigating PanNENs are limited, and technology is still developing [41,48,59].

Genetic fidelity between PDOs and their matched tumors was assessed through diverse molecular profiling approaches, such as STR analysis, WGS, WES, and a targeted gene panel. STR analysis provides the most rapid and cost-effective method to verify that the PDO originated from the corresponding patient tumor. More comprehensive approaches such as WES and WGS offer a higher-resolution view of tumor representation but require greater resources and bioinformatic support. As an intermediate approach, targeted sequencing panels such as TSO500 provide a practical compromise, capturing clinically relevant mutations and copy-number variations with lower costs and data burden. Together, these approaches highlight a trade-off between analytical depth and feasibility, underscoring the need for standardized strategies for PDO validation in future studies.

Among unsuccessful PDO attempts reported in the studies, the majority were observed in PanNET G2. Intermediate-grade tumors are more differentiated and slower growing than G3, which may reduce their adaptability to ex vivo culture conditions. More aggressive tumors (i.e., G3 and NECs) may indeed require less suitable conditions to survive in medium-supported monocultures. This has also been reported as a finding in pancreatic cancer [46,60]. A similar pattern with higher establishment success in high-grade vs. low-grade tumors has also been reported in other cancer types [61]. Furthermore, cells of higher differentiation may also depend more on stromal interactions, which are often lost in conventional PDO monocultures. Some studies retained stromal cells and immune cell components in their culture and generally reported more consistent PDO establishment. Kawasaki et al. [56], who had the highest reported G2 PDO loss, lacked TME preservation, highlighting the potential influence of niche support.

Take rates and culture durability varied widely, with several groups achieving long-term propagation, while others reported low efficiency. These discrepancies likely reflect differences in tissue quality, tumor subtypes, and culture protocols. Matrigel composition, growth factor supplementation, and other culture parameters are known to directly influence take rates and long-term stability. Therefore, the development and adoption of standardized protocols will be essential to improve reproducibility and scalability across studies. Of note, GEP-NET-derived PDOs have been notoriously difficult to maintain in cultures [48], with a previous paper on NET models for ex vivo studies reporting that only 4–12% of cultures have been growing beyond five passages or longer than 6 months compared to other PDOs [62].

In rare tumors such as PanNENs, scarce patient tissue and inconsistent culture success limit reproducibility and constrain the translational potential of the PDO model. Other challenges associated with this model is the increased risk of contamination in prolonged culture periods, and components such as growth factors and 3D matrices required for organoid maintenance contribute to high costs. Ethical considerations also arise from the use of patient-derived material, including informed consent, data privacy, and equitable access to the resulting models.

Beyond these technical and ethical considerations, several sources of bias in PDO research should be acknowledged. Underreporting of unsuccessful PDO establishment may introduce selection bias in the take rates reported (i.e., a bias towards higher take rates than achievable), and a lack of standardized methods such as variations in media composition could lead to performance bias across studies. Experimental variability, including differences in organoid size, passage number, or timing of assessment, may further contribute to measurement bias. Publication bias also remains a concern due to the frequent emphasis on positive findings. Finally, incomplete reporting of key methodological details, such as medium composition or passage history, can compromise reproducibility and limit comparability across studies. Addressing these through transparent reporting will be essential to advance PDOs towards robust and reliable translational applications.

Drug screening represents one of the most clinically promising applications for PDOs [36,38,63,64]. The PDOs in the identified studies were able to capture inter-patient heterogeneity, demonstrated predictive associations with patient outcomes, and identified novel therapeutic vulnerabilities, including EZH2 dependency, PI3K/CDK4/6 synergy, and Bcl-2-linked sensitivities, highlighting PDOs as potential tools for personalized medicine. However, most findings remain preliminary and require validation in other cohorts.

Other areas of interest include the co-culturing of other cellular components and exploring immune-cell function to interrogate the tumor microenvironment and parts of the extracellular matrix that normally surrounds the neoplastic cells [35,65,66].

A strength of this review is its focused scope on PanNENs, providing the first targeted synthesis of studies on PanNEN-derived organoids. This focus distinguishes it from prior PDO reviews that broadly encompass multiple tumor types. By examining PDO generation directly from patient material, it provides a disease-specific perspective on feasibility, fidelity, and translational relevance unique to PanNENs. The systematic methodology, comprehensive literature search, and structured extraction of establishment protocols, validation methods, and drug screening approaches provide a consolidated overview of the current field. In addition, by synthesizing results across both PanNET and PanNEC models, this review highlights shared challenges and unique opportunities in developing PDOs from distinct subtypes.

Some limitations must be acknowledged in this review: First, the overall number of eligible studies was small, reflecting the rarity of PanNENs and the emerging nature of PDO technology. Second, heterogeneity in study design, reporting standards, and validation methods limited the direct comparison across studies. Third, publication bias cannot be excluded, as successful PDO establishment is more likely to be reported than failed attempts. Finally, many studies lacked longitudinal patient correlation, restricting conclusions about the predictive value of PDO drug screening for clinical outcomes.

Together, these strengths and limitations emphasize that while PDOs hold considerable promise as translational models for PanNENs, caution is required in interpreting the current evidence base. Future studies with standardized protocols, transparent reporting of both successes and failures, and integration with clinical endpoints will be essential to validate their utility [63]. Advancing the field will also require integration with single-cell technologies to better characterize tumor heterogeneity and the development of co-culture systems to capture stromal and immune interactions. Emerging technologies such as organoid-on-a-chip devices offer improved control over the microenvironment, with the potential to enhance reproducibility and scalability for translational applications [67]. Although still in early development and not yet standardized, as these systems mature, they may become powerful complements to conventional PDOs in translational and clinical contexts.

## 5. Conclusions

This systematic review provides the first comprehensive synthesis of research into PDOs in PanNETs. Existing studies cover the early experience from 2020 for this tumor entity and demonstrate that PDOs can be established from PanNETs and PanNECs, can retain key histological and molecular features, and can support functional testing including drug screening. PDOs hold promise as translational tools for individualized therapy in this rare cancer type. To realize this potential, future efforts must prioritize protocol standardization, expansion of PanNEC models, and clinical validation of drug response predictions.

## Figures and Tables

**Table 1:** Study characteristics (*n* = 12).

Characteristics	Number
**Publication Period:**	
2020–2022	5
2023–2025	7
**Geographical Distribution:**	
North America	2
Europe	8
Asia	3

**Table 2:** Overview of the studies identified in the systematic review on PanNEN organoid studies. Reported parameters include tumor type, sample origin, PDO establishment rate, maximum reported culture duration, methods of validation and characterization, and functional assays performed.

1st Author (Year, Country)	Tumor Type	Sample Origin	PDO Take Rate	Culture Duration	Validation/ Characterization	Functional Assessment
**Ji (2025, China) [21]**	NF-PanNET (G2-3)	-	4/4	Short-term (>6 days)	H&E, IHC, and Nano-LC-MS/MS	No
**Roalsø (2024, Norway) [46]**	1 PanNEC	Primary tumor	1/3	Short-term (>14 days)	H&E and STR profiling	No
**Strnadel (2024,** **Slovakia/USA) [47]**	PanNET (G3)	Liver met	1/1	Long-term (>20 passages)	H&E, ICC, WB, FACS, confocal imaging, EM, STR profiling, and protein microarray	Xeno
**Wang (2024,** **China) [48]**	NF-PanNET (G3)	Primary tumor	1/1	Long-term (>7 passages)	H&E, IHC, and WES	Tumorigenicity test
**April-Monn (2024,** **Switzerland) [49]**	2 PanNET (G3), 1 PanNEC	2 liver met; 1 LN met	3/3	Short-term (~2 weeks)	H&E, IHC, Targeted panel seq, and RNA-seq	Metabolic Activity and transcriptional profiling
**Alcala (2024,** **France) [50]**	PanNEC ^1^	Primary tumor	1/1	Long-term (>1 year)	H&E, IHC, WGS, and RNA-seq	No
**Dayton (2023,** **Netherlands) [51]**	PanNEC	Primary tumor	1/1	Long-term (>1 year)	H&E, IHC, WGS, and RNA-seq	Viability assay and xeno
**Gulde (2022,** **Germany) [52]**	PanNET	2 primary tumors; 2 liver met	4/4	Short-term (>12 days)	H&E and IHC	Viability assay
**April-Monn (2021,** **Switzerland) [53]**	5 PanNET (G2) 1 PanNEC	4 primary tumors; 2 liver met	6/6	Short-term (>10 days)	H&E and IHC	Viability assay
**Gillette (2021,** **USA) [54]**	PanNET (G2)	Primary tumor	2/2	Short-term (1–2 months)	H&E and IHC	OMI
**April-Monn (2021,** **Switzerland) [55]**	NF-PanNET (G1-2)	5 primary tumors; 1 liver met	6/7	Short-term (≤15 days)	H&E and IHC	Viability assay
**Kawasaki (2020,** **Japan) [56]**	1 PanNET (G3) 2 PanNEC	Primary tumor	3/8	Long-term (>6 months)	H&E, IHC, WGS, WES, RNA-seq, ATAC-seq, and methylation microarray	Xeno and genetic engineering
**Collective sum**			**33/44 (75%)**			

^1^ This PDO line was reported in another publication [51].

**Table 3:** Functional drug screening studies using PDOs from PanNENs. This table summarizes experimental drug testing performed on PanNEN organoids across eligible studies, including sample origin, drug panels, and major findings. Reported PDO assays have been used to evaluate sensitivity to standard-of-care agents as well as targeted or experimental compounds, which are sometimes linked to biomarker discovery.

1st Author (Year)	PDOs Tested	Sample Origin	Drug Panel (Summary)	Key Findings
Ji (2025) [21]	4 NF-PanNETs (G2-3)	Primary tumor	*Preliminary screening:* 10 C2-upregulated targets + 30 FDA approved drugs. *Secondary screening:* 6 hits + 4 PanNET clinical drugs.	Subtype-directed combos targeting C2-upregulated proteins (CACNA1D/CDK5); improved PDO efficacy and reduced PDX tumor growth.
April--Monn (2024) [49]	2 PanNETs (G3); 1 PanNEC	2 liver met; 1 LN met	Cisplatin; temozolomide; IFNB1b; KDM5Ai (CPI-455)	High-grade PanNEN organoids mirrored clinical responses and revealed KDM5A/IFNB1 as cisplatin-sensitizing targets.
Dayton (2023) [51]	1 PanNEC	Primary tumor	Paclitaxel; everolimus (mTOR); navitoclax (Bcl-2); FK866 (NAMPT)	PanNEC organoids showed strong sensitivity towards navitoclax and identified ASCL1 as a potential biomarker for treatment responses.
Gulde (2022) [52]	PanNET	2 primary tumors, 2 liver met	Buparlisib (PI3K) and ribociclib (CDK4/6), tested alone and in combination	Buparlisib + ribociclib combined increased efficacy in PanNET PDOs and in PanNET mice models.
April--Monn (2021) [53]	5 PanNETs (G2); 1 PanNEC	4 primary tumors; 2 liver met	GSK126 (EZH2)	High EZH2 expression observed; EZH2 inhibition active in a subset of PanNEN organoids.
Gillette (2021) [54]	1 PanNET (G2)	Primary tumor	Navitoclax (Bcl-2) ± everolimus (mTOR)	OMI enabled single-cell metabolic measurements of responses to navitoclax and everolimus in live PanNET organoids.
April--Monn (2021) [55]	6 NF-PanNETs (G1-2)	5 primary tumors; 1 liver met	Sunitinib (RTKs); everolimus (mTOR); temozolomide	Establishment of a PanNET drug screening platform with PDOs.

## Data Availability

The data generated or analyzed during this study are included in this published article or are available from the corresponding author upon reasonable request.

## Abbreviations

The following abbreviations are used in this manuscript:

ASCL1	Achaete-scute family bHLH transcription factor 1
ATAC-seq	Assay for transposase-accessible chromatin sequencing
Bcl-2	B-cell lymphoma 2
CACNA1D	Calcium voltage-gated channel subunit alpha1 D
CDK	Cyclin-dependent kinase
CDK4/6	Cyclin-dependent kinase 4/6
CGA	Chromogranin A
CPI-455	KDM5A inhibitor (lysine demethylase 5A inhibitor)
C2	Subtype classification (proteomic signature)
EM	Electron microscopy
EZH2	Enhancer of Zeste homolog 2
FACS	Fluorescence-activated cell sorting
FDA	U.S. Food and Drug Administration
FK866 (NAMPT)	Nicotinamide phosphoribosyltransferase inhibitor
G1–G3	Tumor grades 1 to 3
GEP-NEN	Gastroenteropancreatic neuroendocrine neoplasm
GSK126	GSK2816126 (EZH2 inhibitor compound)
H&E	Hematoxylin and eosin
IHC	Immunohistochemistry
ICC	Immunocytochemistry
IFNB1 (IFN-β1)	Interferon beta 1
INSM1	Insulinoma-associated protein 1
KDM5A	Lysine-specific demethylase 5A
KDM5Ai	Lysine-KDM5A inhibitor
LN	Lymph node
Met	Metastasis
mTOR	Mechanistic target of rapamycin
Nano-LC-MS/MS	Nano-liquid chromatography tandem mass spectrometry
NAMPT	Nicotinamide phosphoribosyltransferase
NEC	Neuroendocrine carcinoma
NEN	Neuroendocrine neoplasm
NET	Neuroendocrine tumor
NF-PanNET	Non-functional pancreatic neuroendocrine tumor
OMI	Optical metabolic imaging
OS	Overall survival
PanNEC	Pancreatic neuroendocrine carcinoma
PanNEN	Neuroendocrine neoplasm
PanNET	Pancreatic neuroendocrine tumor
PDO	Patient-derived organoid
PDX	Patient-derived xenograft
PI3K	Phosphoinositide 3-kinase
PICO	Population, intervention, comparison, and outcome (systematic review framework)
PRISMA	Preferred Reporting Items for Systematic Reviews and Meta-Analyses
PRRT	Peptide receptor radionuclide therapy
RNA-seq	RNA sequencing
RTK	Receptor tyrosine kinase
SSA	Somatostatin analog
SSTR2	Somatostatin receptor 2
STR	Short tandem repeat
SYN	Synaptophysin
VEGF	Vascular endothelial growth factor
WB	Western blot
WES	Whole-exome sequencing
WGS	Whole-genome sequencing
Xeno	Xenotransplantation
